# An EEG investigation of alpha and beta activity during resting states in adults with Williams syndrome

**DOI:** 10.1186/s40359-021-00575-w

**Published:** 2021-05-05

**Authors:** Joanna M. H. Greer, Deborah M. Riby, Mhairi E. G. McMullon, Colin Hamilton, Leigh M. Riby

**Affiliations:** 1 Department of Psychology, Northumbria University, Newcastle upon Tyne, UK; 2 Department of Psychology, Durham University, Durham, UK; 3 Centre for Developmental Disorders, Durham University, Durham, UK

**Keywords:** Williams syndrome, EEG, Alpha, Beta, Attention, Inhibition

## Abstract

**Background:**

Williams syndrome (WS) is neurodevelopmental disorder characterised by executive deficits of attention and inhibitory processing. The current study examined the neural mechanisms during resting states in adults with WS in order to investigate how this subserves the attention and inhibitory deficits associated with the syndrome.

**Method:**

Adopting electroencephalography (EEG) methodology, cortical electrical activity was recorded from eleven adults with WS aged 35 + years during Eyes Closed (EC) and Eyes Open (EO) resting states, and compared to that of thirteen typically developing adults matched for chronological age (CA) and ten typically developing children matched for verbal mental ability (MA). Using mixed-design analyses of variance (ANOVA), analyses focused on the full alpha (8–12.5 Hz), low-alpha (8–10 Hz), upper-alpha (10–12.5 Hz), and beta (13–29.5 Hz) bands, as these are thought to have functional significance with attentional and inhibitory processes.

**Results:**

No significant difference in alpha power were found between the WS and CA groups across all analyses, however a trend for numerically lower alpha power was observed in the WS group, consistent with other developmental disorders characterised by attentional/inhibitory deficits such as Attention Deficit Hyperactivity Disorder (ADHD). In contrast, comparable beta power between the WS and CA groups during both EC/EO conditions suggests that their baseline EEG signature is commensurate with successful attentional processing, though this needs to be interpreted with caution due to the small sample size. Analyses also revealed an unusual trend for low variability in the EEG signature of the WS group, which contradicts the heterogeneity typically observed behaviourally.

**Conclusions:**

This novel finding of low variability in the EEG spectra in the WS group has been previously associated with poor behavioural performance in ADHD and is highly informative, highlighting future research needs to also consider how the role of low variability in the EEG profile of WS manifests in relation to their behavioural and cognitive profiles.

## Background

Williams syndrome (WS) is a neurodevelopmental disorder with an estimated prevalence of 1:18,000 [1], caused by a micro-deletion of approximately 28 genes on chromosome 7 (7q11.23) [2]. Several candidate genes (e.g. LIMK1, CYLN2, GTF21) are known to have neuronal expression relevant to the behavioural and cognitive phenotype associated with WS, and which draw attention as specific genetic markers of syndrome (see [3] for a review). Despite the heterogeneity of cognitive ability [4], individuals with WS typically function at the level of mild-to-moderate intellectual difficulty [5]. Cognitive scientists are drawn to WS in particular due to the distinctive cognitive profile associated with the syndrome. Relatively more impaired visuo-spatial skills (e.g. [6, 7]) compared with relatively less impaired verbal processing [8] are widely documented in the literature, though always within their level of intellectual difficulty. Despite the parallel of heterogeneity in the cognitive and behavioural profiles associated with WS [9], many individuals with WS are exceptionally sociable in nature, are eager to engage in conversation with others, and have a propensity to approach strangers at random [10, 11].

Evidence from a growing body of research now emphasises that the behavioural, cognitive, and social profiles associated with WS are grounded in impairments in the executive functioning processes of attention and inhibition (e.g. [9, 12, 13]). Recent research adopting event-related potential (ERP) methodology has been informative in highlighting atypicalities in the ERP signature in WS, linking this to known atypicalities in the behavioural and cognitive phenotype of the syndrome (e.g. [14–19]). However there are inconsistencies in the ERP literature from other developmental disorders (e.g. attention-deficit hyperactivity disorder (ADHD) [20]; Fragile X syndrome (FXS) [21], likely reflecting the recruitment of less impaired/spared cortical and subcortical regions in order to achieve the same behavioural result. Certainly, it has been reported in many areas of functioning and across the WS developmental spectrum, that seemingly good performance might be achieved by ‘different’ routes and using different mechanisms (e.g. face perception; [22]). In light of this, the focus of this study was to investigate baseline cortical activity in adults with WS, in the absence of goal-directed cognitive processing. The central aim of the current study was to identify any differences in the resting-state neural signature which may underpin the attentional and inhibitory behavioural profiles associated with the syndrome. In order to provide a more comprehensive profile of neuropsychological processes in WS, the current study adopted electroencephalography (EEG) methodology in order to observe cortical activity in the alpha and beta bands during resting states, and elucidate how these profiles sub-serve the behavioural and cognitive phenotypes associated with the syndrome.

### Functional significance of the alpha and beta bands

The alpha band is primarily associated with attention, inhibitory processes, and the mechanisms of attention and consciousness (for a review, see [23]). Unlike the other frequency bands, alpha activity reports an inverse profile whereby an increase in alpha power (synchronisation) is indicative of less cortical activity, whilst a decrease in alpha power (desynchronisation) reflects activity in response to visual/sensory input [24–27]. This increased alpha power is believed to reflect cortical inhibitory processes, whereas decreased alpha power reflects a release from cortical inhibitory control, enabling the recruitment of attentional resources in response to changing task demands [28, 29]. Atypical patterns in alpha synchronisation/desynchronisation are functionally associated with impairments in cognitive processing. For example, during stimulus–response tasks such as the three-stimulus Oddball paradigm [30], decreased alpha power prior to an upcoming *No-Go* stimulus signals a release from cortical inhibition thus enabling the cognitive processes required for inhibiting motor actions [31, 32]. However, brief increases in pre-frontal and posterior pre-stimulus alpha power are associated with attentional lapses and poorer task performance [33, 34].

Likewise, the role of the beta band in top–down visual-attentional processing is widely documented (e.g. [35–37]). Occipito-parietal beta power is associated with better performance on tasks which recruit attentional processes (e.g. [36, 38, 39]), whilst increases and decreases in beta power are also functionally associated with the execution and inhibition of voluntary movements. During response inhibition tasks, increased pre-stimulus beta power predicts successful inhibition in response to *No-Go* stimuli [40, 41]. In contrast, beta activity post-commission errors is characterised with greater rebound, indicative of increased response inhibition [42].

The functional roles of the alpha and beta bands are supported by behavioural deficits which can be linked to atypicalities in alpha and beta activity. A recent study investigating age-associated differences in beta activity during a sustained attention task in typically developing (TD) adults [43] found a sub-group of lower performing older adults, identified by greater behavioural deficits in sustained attention, reported significantly attenuated beta activity when more demanding attentional processing was required. Furthermore, this group also reported increased alpha activity with greater task difficulty, indicative of impaired task-specific alpha desynchronisation [44].

Similarly, the literature on developmental disorders supports the link between alpha/beta dysfunction and deficits in executive processes of attention and inhibition. Due to the lack of EEG research in WS, inspection of the EEG profile found in ADHD is informative as there are notable similarities in the attentional and inhibitory profiles between the two groups [45–47]. During a continuous attention performance task (CPT), adults with ADHD displayed significantly lower frontal power in the low-alpha sub-band (8–10 Hz) and greater beta power compared with healthy age-matched TD controls, indicative of increased cortical activity during sustained attention [48]. Furthermore, despite comparable behavioural performance, low-alpha was attenuated for the duration of the task in the ADHD group but gradually increased in the control group, indicative of lesser reliance on the inhibitory function of alpha during sustained attention across time. Loo and colleagues [48] also found significant correlations between frontal low-alpha power and increased commission errors/decreased reaction time (RT) in their controls but not the ADHD adults, indicative of an association between increasing low-alpha and impulsive response profile in TD individuals. In contrast, the ADHD group only reported a significant negative correlation of increased beta power and decreased behavioural task variability. Thus, chronic attenuated low-alpha and enhanced beta power in ADHD appears to be a compensatory mechanism, notably with increasing task demands, whereby this group require greater cortical activity to maintain sustained attention and reduce behavioural variability. This emphasises the need to include electrophysiological measures alongside behavioural paradigms in research with individuals who have developmental disorders.

### Resting states—eyes closed/eyes open

Thus far, we have emphasised the role of the alpha and beta bands during goal-directed cognitive processing in typical and atypical development. However, as demonstrated in the neurodevelopmental literature, under certain task conditions, atypically developing groups can perform as well as TD individuals (behaviourally) (e.g. [22]). Thus, elucidating how and why the neural mechanisms and their associated behavioural processes differ between individuals with developmental disorders and those developing typically can be problematic. Electrophysiological activity whilst unconscious (i.e. during sleep/coma) and during resting states (i.e. relaxed conscious) presents distinct profiles that can be dissociated from conscious sensory and cognitive processing [49–51]. By studying neural activity in the absence of stimulus-induced/goal-directed activity, researchers can distinguish how cortical and subcortical processes differ between active and passive conditions.

Resting-state activity is typically recorded by implementing Eyes Closed (EC; whereby participants rest with their eyes closed), and/or Eyes Open (EO; where they focus on a non-task-related visual stimulus) paradigms. During an EC resting state, both alpha and beta report synchronised activity which is typically distributed over parieto-occipital regions [52]. Importantly, EEG sub-bands report different EC profiles. Low-alpha reports a more widespread topography across anterior–posterior regions, whereas upper-alpha and beta are dominant posteriorly. Opening the eyes results in topographic changes whereby both alpha and beta bands demonstrate attenuation in power. However, the decreases in posterior regions are more pronounced in alpha, whereas beta reports smaller posterior decreases and pre-frontal increases, believed to be the engagement of frontally controlled regions responsible for executive processes [52–55].

Research with developmental disorders highlights atypicalities in the resting-state EEG profile. For example, during five minutes of EC, Babiloni et al. [56] observed significantly attenuated alpha, beta, and gamma in adolescents with Down syndrome (DS) compared to a TD chronologically age-matched (CA) control group. Likewise, Woltering, Jung, Liu, and Tannock [57] observed attenuated alpha power in participants with ADHD compared to controls during both EC and EO, whilst attenuated beta is widely acknowledged in the atypical theta/beta ratio [58]. In FXS, comparable beta power has been observed between these individuals and TD CA-matched controls, but significantly attenuated in the upper-alpha band (10–12.5 Hz) [59], and is linked to executive dysfunction such as attentional lapses (cf. WS; [60]). In contrast, there are mixed findings in the EEG resting-state profile in autism spectrum disorder (ASD) (for a review, see [61]).

### EEG profile in Williams syndrome

In the WS literature, the focus on neuroimaging methods such as fMRI (functional Magnetic Resonance Imaging) and EEG is notably lacking compared to other developmental disorders such as ASD and ADHD. However, from the available literature, it would appear that an atypical EEG profile is present in WS under certain conditions, and which is in line with other developmental disorders [15, 62–66].

To date there is only one known study that specifically focuses on the EEG signature in WS during conscious resting states. Ng, Fishman, and Bellugi [67] investigated the profile of the alpha band in a combined EC/EO paradigm in a cohort of adults with WS (n = 9) and a group of TD controls (not matched for either CA or mental (MA) age). Of specific interest to the authors were frontal inter-hemispherical resting-state differences, which might underpin the disinhibited social profile associated with the syndrome. The WS group reported attenuated frontal alpha power in the left hemisphere, but no group differences in the right hemisphere. Notably, the WS and TD group demonstrated an opposite pattern of intra-hemispheric asymmetry. The WS group reported greater right over left hemispherical asymmetry, whereas the TD controls reported greater left over right asymmetry. Ng and colleagues [67] functionally associate the over-recruitment of the left hemisphere in their WS group with a neuropsychological profile including exaggerated anxieties associated with the syndrome [68, 69]. As the EC and EO data were combined in the analysis, it is not possible to interpret the functional significance of their results between the two resting states. However, their study is highly informative, as the under-recruitment of the right frontal hemisphere in the WS group provides preliminary evidence for atypical baseline activity during resting states in WS in the cortical regions functionally associated with inhibitory processes (but also see [70]).

### Hypotheses

The aim of the current study was to characterise the alpha and beta band EEG profiles in adults with WS during both Eyes Closed and Eyes Open resting states. The three groups that made up the participants for this study were adults with WS (aged 35+ years), TD adults matched for chronological age (CA), and TD children matched for verbal mental ability (MA). We have previously reported findings from behavioural and electrophysiological studies which have included a verbal MA-matched control group [14, 71]. In light of the ongoing debate in the developmental disorder literature regarding group matching with TD children (see [72]), we have included comparison with this MA-matched group in order to highlight differences in the electro-cortical profiles between adults with WS (i.e. atypical but developed neuronal maturation) and TD children with ongoing neuronal maturation. In light of the dearth of EEG research with WS, hypotheses have been primarily guided by the ADHD literature due to the neurocognitive similarities between the two syndromes [46, 47]. It was hypothesised that adults with WS would report overall attenuated alpha (full alpha and both sub-bands) compared to the controls in both the Eyes Closed and Eyes Open conditions, reflective of the suggested state of hyper-cortical arousal as found in ADHD. Attenuated beta power in WS was also hypothesised in both Eyes Closed and the Eyes Open conditions, reflective of the attentional deficits observed in their behavioural profile. Overall greater power values across all frequencies of interest was hypothesised in the MA group’s EEG profile reflecting their developmental stage of neuronal maturation.

## Method

### Participants

The sample consisted of three groups; adults with Williams Syndrome (WS), typically developing adults matched for chronological age (CA), and typically developing children matched for verbal mental ability (MA). The WS group consisted of eleven adults (7 males, aged 37 years 2 months–49 years 3 months, mean age 42 years 7 months, SD 48 months) recruited via the Williams Syndrome Foundation, UK. Fluorescence in situ hybridisation (FISH) testing had previously confirmed the genetic diagnosis for nine participants, whilst diagnosis for two participants was based on their clinical phenotype. Four of the WS participants lived independently, and seven lived in the parental home or sheltered accommodation supported by carers. Six were employed or undertook volunteer work, and the remainder attended daycare centres or received care assistance provided by their local authority.

Sixteen typically developing adults matched for chronological age were recruited for the CA group (9 males, aged 36 years 10 months–49 years 2 months, mean age 42 years 10 months, SD 50 months). Thirteen typically developing children were recruited for the MA group (7 males, aged 8 years 7 months–15 years 7 months, mean age 12 years 2 months, SD 32 months). WS/MA group matching was based on receptive vocabulary using the raw scores (mean: WS—116.82, SD 10.36; MA—117.54, SD 12.98) from the British Picture Vocabulary Scale (BPVS-II; [73]). Exclusion criteria for the CA and MA groups were a diagnosis of any developmental disorder (e.g. ASD and ADHD). Where possible, written informed consent was provided by participants in the WS group, and was provided by parents/carers of all participants in the WS and MA groups.

The Edinburgh Handedness Inventory (EHI) was used to assess handedness in all participants [74]. All CA and MA participants were right-handed, whilst four of the WS group were left-handed. Participants in the CA/MA groups received £6.00 for participating in the study. Ethical approval was obtained from the Department of Psychology ethics committee, Northumbria University.

### Physiological recording

Data collection for all participants took place in the participants’ place of residence or in the Psychology Department, Northumbria University. Parents/carers of the WS and MA participants were present at the session or nearby. After the experimental procedure was explained, participants were invited to read and sign the informed consent form and complete the EHI.

Continuous EEG was recorded from 32 channels comprising of 4 midline sites (FZ, CZ, PZ, OZ), 14 left hemisphere sites (Fp1, AF3, F3, F7, Fc1, Fc5, C3, T7, Cp1, Cp5, P3, P7, Po3, O1), and 14 right hemisphere sites (Fp2, Af4, F4, F8, Fc2, Fc6, C4, T8, Cp2, Cp6, P4, P8, Po4, O2). Electrode placement was based on the extended international 10–20 system [75] using an electrode cap (Biosemi, Amsterdam, the Netherlands). The CMS/DRL electrodes were placed adjacent to the CZ electrode forming a virtual ground, with average electrode referencing computed offline. Eye blinks were assessed with electrodes placed above and below the left eye to record the vertical electrooculogram.

Power estimates were derived from the average for full-alpha (8–12.5 Hz), low-alpha (8–10 Hz), upper-alpha (10–12.5 Hz), and beta (13–29.5 Hz) frequency bands at frontal (F3, FZ, F4), central (C3, CZ, C4), and parietal (P3, PZ, P4), and occipital (O1, OZ, O2) sites (see [48]).

### Procedure

The participants were advised they would be required to sit still with their eyes closed for 2 min, then sit still with their eyes open for a further 2 min. During both conditions, the participants were asked to remain relaxed and silent, avoid head and body movements, and refrain from blinking if possible. During the Eyes Open procedure, the participants were instructed to focus on a neutral spot straight ahead of them, and avoid eye movements for the duration of the task.

### Data extraction

Prior to data collection, as an estimate of electrode noise, Biosemi electrode offset values were considered. Values between − 20 and + 20 mV were considered good. Automatic eye blink correction, artefact rejection (values outside the range of − 100 to + 100 μV), and EEG averaging were carried out off-line using Neuroscan SCAN 4.5 software (Compumedics, El Paso, TX). The EEG data from each 2-min segment were divided into 2-s epochs from the start of data recording. Each epoch was subject to visual inspection and any epochs containing artefacts such as eye movements and blinks were manually rejected. For each subject in both conditions, average power spectra were calculated using Fast Fourier Transforms. At each electrode, absolute power in full alpha (8–12 Hz), low-alpha (8–10 Hz), upper-alpha (10–12.5 Hz) and the beta (13–29.5 Hz) bands were calculated.

Data from three from the CA group, and three from the MA group were excluded due to EEG artefacts which compromised further analysis. Thus the final sample consisted of eleven adults with WS, thirteen adults matched for chronological age (CA), and ten children matched for verbal mental ability (MA).

## Results

In order to characterise the resting state activity in our populations we focused on the dominant alpha and beta spectra in the brain. We present here analyses of Eyes Closed and Eyes Open separately to capture different electrophysiological components related to arousal and attention at rest (see Tables 1, 2). To explore these differences, a series of analyses of variance (ANOVA) were employed to investigate group effects (WS vs. CA vs. MA) in EEG power and importantly variability in scalp topography (hemispheres—left vs. midline vs. right; location—frontal vs. central vs. parietal vs. occipital). The ANOVAs were conducted on the following frequencies a: alpha (*α-full*), 8–12.5 Hz, b: low-alpha (*α-low*), 8–10 Hz, c: upper-alpha (*α-high)*, 10–12.5 Hz, and d: beta (β), 13–29.5 Hz. Where Mauchly’s test of sphericity was significantly violated, a Greenhouse–Geisser correction was used. Bonferroni corrected pairwise comparisons were employed to analyse significant main effects. To provide a summary of group effects, and to allow comparison with research endeavours elsewhere, Cohen’s *d* was calculated for the overall difference between the WS individuals and control participants.

**Table 1:** Mean alpha and beta power (SDs in parentheses) by location in the Eyes Closed condition

Band	Location	WS	CA	MA
Full alpha	Frontal	0.98 (0.75)	3.20 (3.51)	6.69 (10.03)
	Central	0.68*^1^ (0.55)	2.10 (2.19)	4.98 (4.57)
	Parietal	1.18*^1^ (1.01)	4.28 (4.67)	12.98 (19.00)
	Occipital	2.84*^1^ (2.28)	11.39 (11.75)	27.91 (26.91)
Low alpha	Frontal	1.59 (1.57)	3.98 (4.63)	7.93 (11.19)
	Central	0.87*^1^ (0.71)	2.56 (3.02)	6.05 (6.58)
	Parietal	1.80*^1^ (2.09)	4.46 (4.94)	16.21 (21.57)
	Occipital	4.57*^1^ (4.36)	14.07 (17.76)	27.51 (21.71)
Upper alpha	Frontal	0.49 (0.24)	2.57 (2.96)	5.70 (9.28)
	Central	0.53*^1^ (0.47)	1.74*^2^ (1.76)	4.13 (3.24)
	Parietal	0.68 (0.46)	4.14 (4.86)	10.39 (17.4)
	Occipital	1.43*^1^ (0.98)	9.25 (8.81)	28.23 (35.05)
Beta	Frontal	0.49 (0.37)	0.37 (0.24)	0.78 (0.54)
	Central	1.22 (2.65)	0.37 (0.28)	0.54 (0.37)
	Parietal	0.50 (0.46)	0.42 (0.27)	0.79 (0.63)
	Occipital	0.96 (1.04)	0.82 ( 0.45)	1.94 (1.02)

^*^^1^Indicates significant difference (in bold) between WS/MA groups (*p* < .05)

^*^^2^Indicates significant difference (in bold) between CA/MA groups (*p* < .05)

**Table 2:** Mean alpha and beta power (SDs in parentheses) by location in the Eyes Open condition

Band	Location	WS	CA	MA
Full alpha	Frontal	0.69*^1^ (0.65)	1.19 (1.69)	2.96 (3.31)
	Central	0.47*^1^ (0.24)	0.81 (0.95)	3.01 (2.67)
	Parietal	**0.72**^**1**^ (0.94)	1.65 (2.58)	4.39 (5.9)
	Occipital	1.21*^1^ (0.91)	2.74 (4.28)	6.17 (6.73)
Low alpha	Frontal	0.98*^1^ (1.11)	1.23 (1.15)	3.71 (3.22)
	Central	0.50*^1^ (0.56)	0.85 (1.04)	3.64 (3.91)
	Parietal	1.11*^1^ (1.71)	1.41 (1.64)	5.74 (7.11)
	Occipital	1.80*^1^ (2.09)	2.45 (3.58)	6.17 (6.44)
Upper alpha	Frontal	0.38*^1^ (0.18)	1.15 (1.83)	2.35 (2.59)
	Central	0.37*^1^ (0.2)	0.78 (0.91)	2.51 (1.69)
	Parietal	0.40*^1^ (0.17)	1.84 (3.2)	3.31 (4.21)
	Occipital	0.75*^1^ (0.29)	2.97 (4.85)	3.20 (5.16)
Beta	Frontal	0.66 (0.6)	0.36 (0.26)	0.75 (0.48)
	Central	0.61 (0.65)	0.30 (0.27)	0.46 (0.32)
	Parietal	0.31 (0.14)	0.31 (0.21)	0.60 (0.47)
	Occipital	0.67*^1^ (0.49)	0.63*^2^ (0.41)	1.32 (0.86)

^*^^1^Indicates significant difference (in bold) between WS/MA groups (*p* < .05)

^*^^2^Indicates significant difference (in bold) between CA/MA groups (*p* < .05)

### Eyes closed

#### Alpha band (α-full): 8–12.5 Hz

There was a significant main effect of group [F(2,31) = 5.466, *p* = 0.009], and location [F(1.458,45.191) = 18.233, *p* < 0.001], on *α-full* power. The main effect of group was due to significantly lower *α-full* power in the WS group compared with the MA group (*p* = 0.008, *d* = 1.18). There was no difference in *α-full* power between the WS and the CA groups (*p* = 0.799, *d* = 0.98), or between the CA versus MA groups (*p* = 0.090, *d* = 3.21). Examining location, Bonferroni corrected pairwise comparisons revealed significantly greater occipital *α-full* power compared with the frontal, central and parietal locations (*p* ≤ 0.003). Parietal *α-full* power was significantly greater than the frontal location (*p* = 0.039) but not the central (*p* = 0.094) location. The difference in *α-full* power between frontal and central locations did not reach significance (*p* = 0.438). These data regarding topography are consistent with the expected parieto-occipital maxima at rest [52].

Significant interactions between location × group [F(2.916,45.191) = 4.912, *p* = 0.005], and hemisphere × location [F(1.242,38.505) = 5.657, *p* = 0.017] were also observed. Exploring the interaction with group, *α-full* power differences were evident at central [F(2,31) = 6.239, *p* = 0.005], parietal [F(2,31) = 3.436, *p* = 0.045], and occipital [F(2,31) = 6.380, *p* = 0.005] locations, primarily driven by greater power in the MA group. Bonferroni corrected pairwise comparisons revealed significantly greater *α-full* power in the MA than the WS group at the central (*p* = 0.005, *d* = 1.32), parietal (*p* = 0.050, *d* = 0.87), and occipital (*p* = 0.004, *d* = 1.31) locations, and numerically greater *α-full* power in the MA group which approached significance compared with the CA group at the central (*p* = 0.065, *d* = 0.80) and occipital (*p* = 0.066, *d* = 0.79) locations. All other comparisons were non-significant (*p* ≥ 0.098). The hemisphere by location interaction was driven by significantly attenuated frontal and central *α-full* power (*p* ≤ 0.003) and significantly greater occipital *α-full* power (*p* < 0.001) compared to the left, midline, and right sites.

The effects sizes between the WS group and the MA and CA where the *α-full* power maxima, at rest, has been reported to be centred (PZ; 53) were *d* = 0.88 (WS vs. MA) and *d* = 0.92 (WS vs. CA).

#### Low-alpha band (α-low): 8–10 Hz

**Fig. 1:**
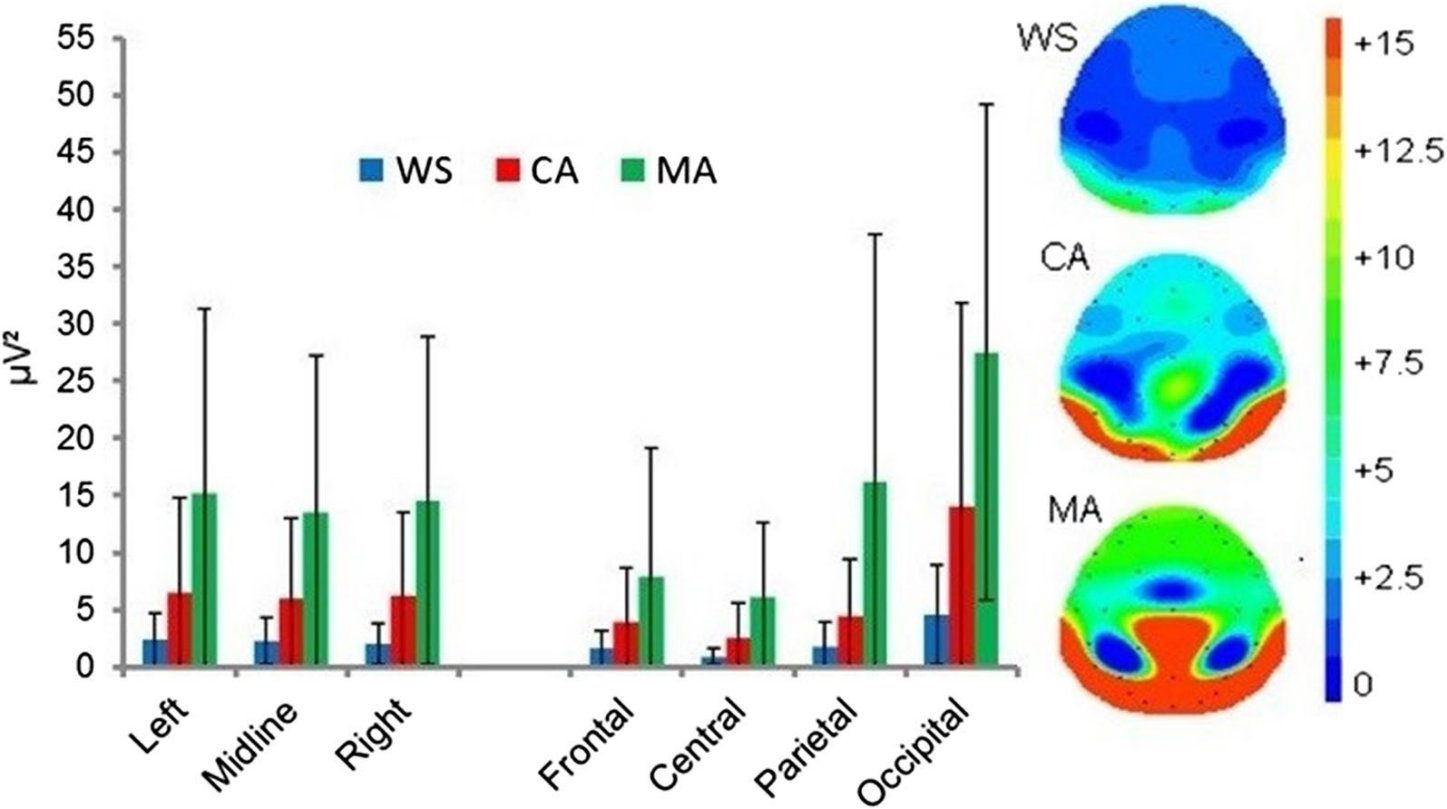
Mean absolute Eyes Cosed low-alpha power (µV^2^) and spectral mapping. Error bars represent SDs

Due to the suggested disparate functions associated with low and upper alpha power [27] and the ratio between the two being related to cognitive dysfunction (e.g. [76]), separate analyses of *Low* and *Upper* are consider here. The ANOVA revealed a significant main effect of group [F(2,31) = 4.754, *p* = 0.016], due to significantly lower *α-low* power in the WS compared with the MA group (*p* = 0.014, *d* = 1.17), but not between the WS versus CA (*p* = 0.872, *d* = 0.75) or the CA versus MA (*p* = 0.130, *d* = 0.70) groups. A significant main effect of location was also observed [F(1.615,50.061) = 22.671, *p* < 0.001] driven by significantly greater occipital *α-low* power compared with frontal, central, and parietal locations (*p* ≤ 0.002). Parietal *α-low* power was also significantly greater than that observed frontally (*p* = 0.036) and centrally (*p* = 0.031). No difference was observed in *α-low* power between the frontal and central locations (*p* = 0.067). See Fig. 1.

Analyses revealed a significant location × group interaction [F(3.230,50.061) = 4.239, *p* = 0.008], The exploration of this interaction revealed group differences in *α-low* power at central [F(2,31) = 4.472, *p* = 0.020], parietal [F(2,31) = 4.216, p = 0.024], and occipital [F(2,31) = 5.219, *p* = 0.011] locations. Bonferroni corrected pairwise comparisons revealed group differences were due to significantly lower *α-low* power in the WS group compared to the MA group centrally (*p* = 0.018, *d* = 1.11), parietally (*p* = 0.031, *d* = 0.94), and occipitally (*p* = 0.009, *d* = 1.46). All other analyses were non-significant (*p* ≥ 0.083).

A significant location × hemisphere [F(1.527,47.324) = 6.452, *p* = 0.006] interaction was observed. Again, the interaction effect was due to significantly attenuated frontal and central, and significantly greater occipital *α-low* power compared with the left, midline, and right sites (*p* < 0.001).

The effects sizes between the WS group and the MA and CA groups where *α-low* power maxima, at rest, has been reported to be centred (PZ; [27]) were *d* = 0.94 (WS vs. MA) and *d* = 0.70 (WS vs. CA).

#### Upper-alpha band (α-high): 10–12.5 Hz

**Fig. 2:**
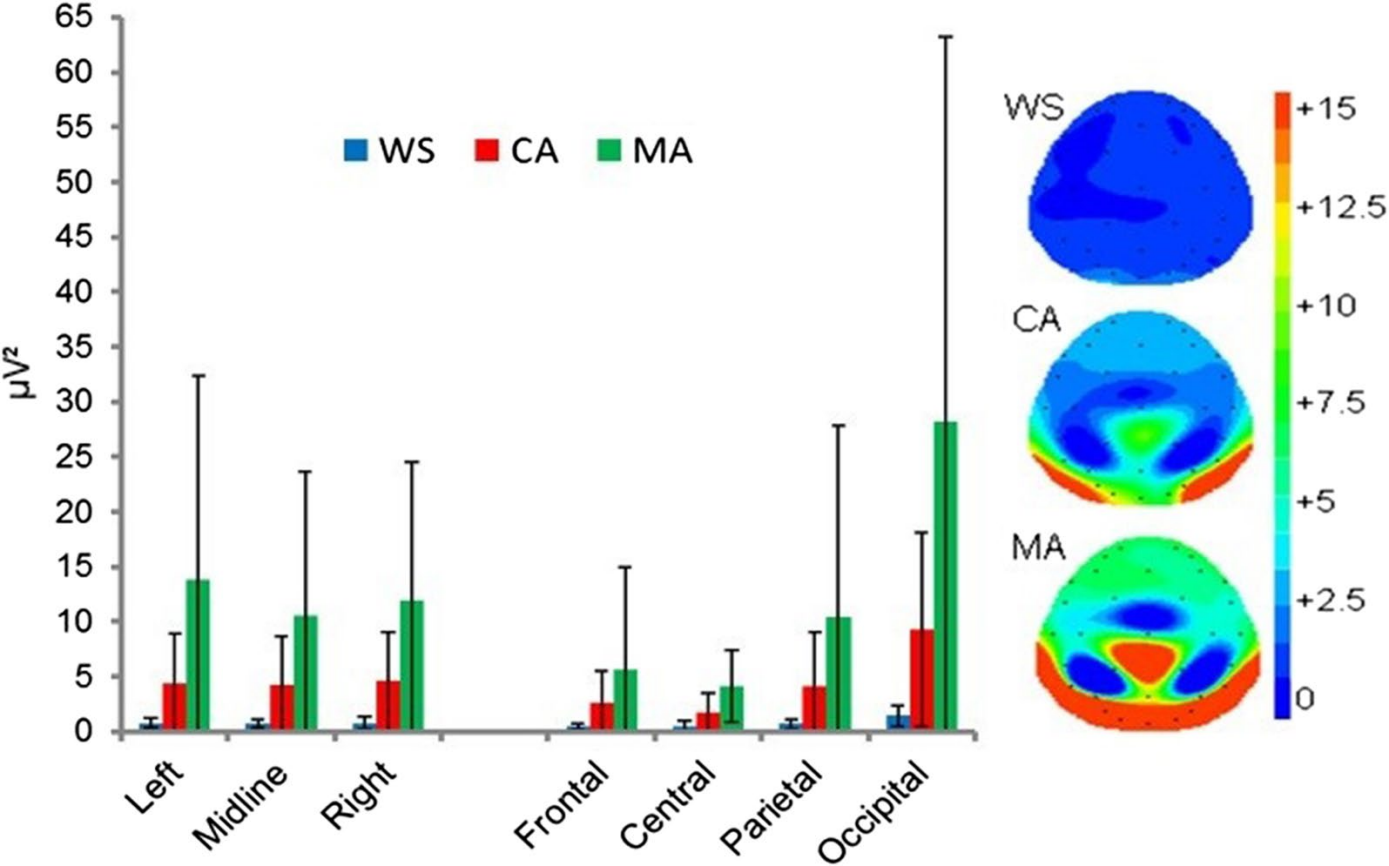
Mean absolute Eyes Closed upper-alpha power (µV^2^) and spectral mapping. Error bars represent SDs

The ANOVA revealed a significant main effect of group [F(2,31) = 4.993, *p* = 0.013]. Bonferroni corrected pairwise comparisons revealed significantly lower *α-high* power in the WS group compared with the MA group (*p = 0*.012, *d* = 1.09). No differences were observed between the WS versus CA groups (*p* = 0.887*, d* = 1.15) or between the CA versus MA groups (*p* = 0.109, *d* = 0.71). The main effect of location [F(1.275,39.523) = 10.435, *p* = 0.001], was due to significantly greater occipital *α-high* power compared with the frontal (*p* = 0.008), central (*p* = 0.010), and parietal (*p* = 0.036) locations. All other analyses were non-significant (*p* ≥ 0.071). See Fig. 2.

The analyses also revealed a significant location × group interaction [F(2.550,39.523) = 3.977, *p* = 0.019]. Exploration of this interaction revealed significant group effects at the central [F(2,31) = 8.104, *p* = 0.001], and occipital [F(2,31) = 5.117, *p* = 0.012] locations only. Bonferroni corrected pairwise comparisons revealed significantly greater central *α-high* power in the MA group compared with both the WS (*p* = 0.001, *d* = 1.56) and CA (*p* = 0.030, *d* = 0.92) groups. Occipital *α-high* power was also significantly greater in the MA group compared with the WS group (*p* = 0.012, *d* = 1.08). All other comparisons were non-significant (*p* ≥ 0.086). A significant location × hemisphere interaction [F(1.252,38.823) = 3.985, *p* = 0.044] was also observed. Paired samples t-tests revealed significantly attenuated *α-high* power at frontal and central locations (*p* ≤ 0.016) and significantly greater occipital *α-high* (*p* ≤ 0.008) compared with the left, midline, and right sites.

The effects sizes between the WS group and the MA and CA groups where *α-upper* power maxima has been reported to be centred (PZ; 27) were *d* = 0.79 (WS vs. MA) and *d* = 1.00 (WS vs. CA).

#### Beta band (β): 13–29.5 Hz

**Fig. 3:**
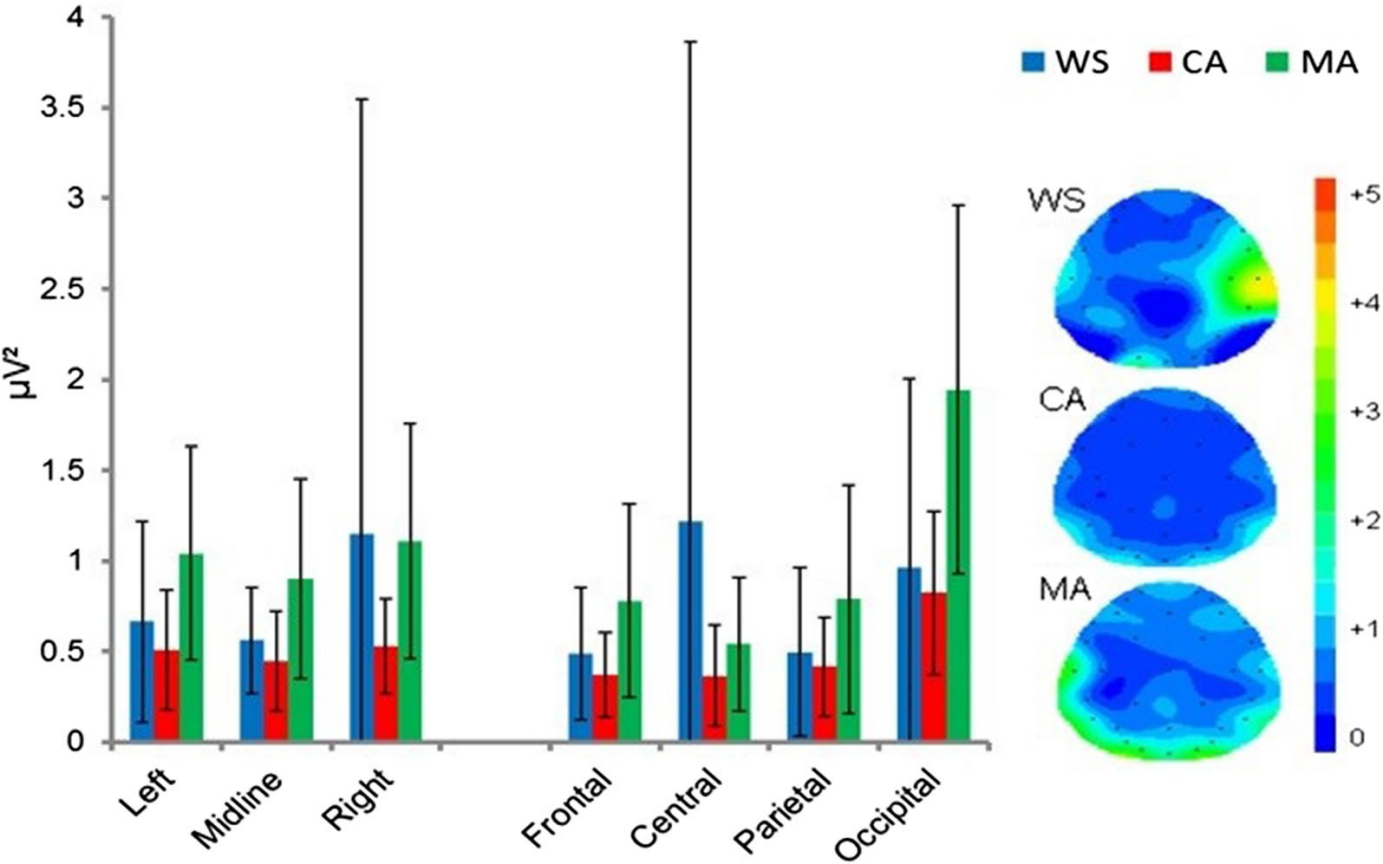
Mean absolute Eyes Closed beta power (µV^2^) and spectral mapping. Error bars represent SDs

The ANOVA examining beta spectra revealed a significant main effect of location [F(1.356,42.042) = 5.781, *p* = 0.013]. Bonferroni corrected pairwise comparisons revealed significantly greater occipital β power compared with the frontal and parietal locations (*p* < 0.001). All other comparisons by location were non-significant (*p* ≥ 0.458). All other main and interaction effects were non-significant (*p* ≥ 0.105). See Fig. 3.

The effects sizes between the WS group and the MA and CA groups where *Beta* power maxima has been reported to be centred (FZ; [77]) were *d* = 0.62 (WS vs. MA) and *d* = 0.39 (WS vs. CA).

### Summary of the Eyes Closed results

The typically reported posterior maxima topographical distribution was observed in the alpha band (full alpha and sub-bands) and beta band in all three groups. Group differences were typically due to significantly greater alpha power in the MA group compared to the WS group; however numerically lower alpha power was consistently observed in the WS group compared to the CA controls. No group differences in beta power were observed.

### Eyes Open

#### Alpha band (α-full): 8–12.5 Hz

Analyses identified a significant main effect of group [F(2,31) = 3.930, *p* = 0.030] on *α-full* power. Bonferroni corrected pairwise comparisons identified significantly greater *α-full* power in the MA group compared with the WS group (*p* = 0.033, *d* = 1.05), but no difference in *α-full* power between the WS versus CA (*p* = 1.00, *d* = 0.049) and CA versus MA (*p* = 0.131, *d* = 0.70) groups. The significant main effect of location [F(1.369,42.432) = 10.444, *p* = 0.001] was due to significantly greater occipital *α-full* power compared with the frontal (*p* = 0.003), central (*p* = 0.011), and parietal (*p* = 0.012) locations. All other main effects and interactions were non-significant (*p* ≥ 0.088).

The effects sizes between the WS group and the MA and CA where the *α-full* power maxima has been reported to be centred (PZ; 53) were *d* = 0.86 (WS vs. MA) and *d* = 0.48 (WS vs. CA).

#### Low-alpha band (α-low): 8–10 Hz

**Fig. 4:**
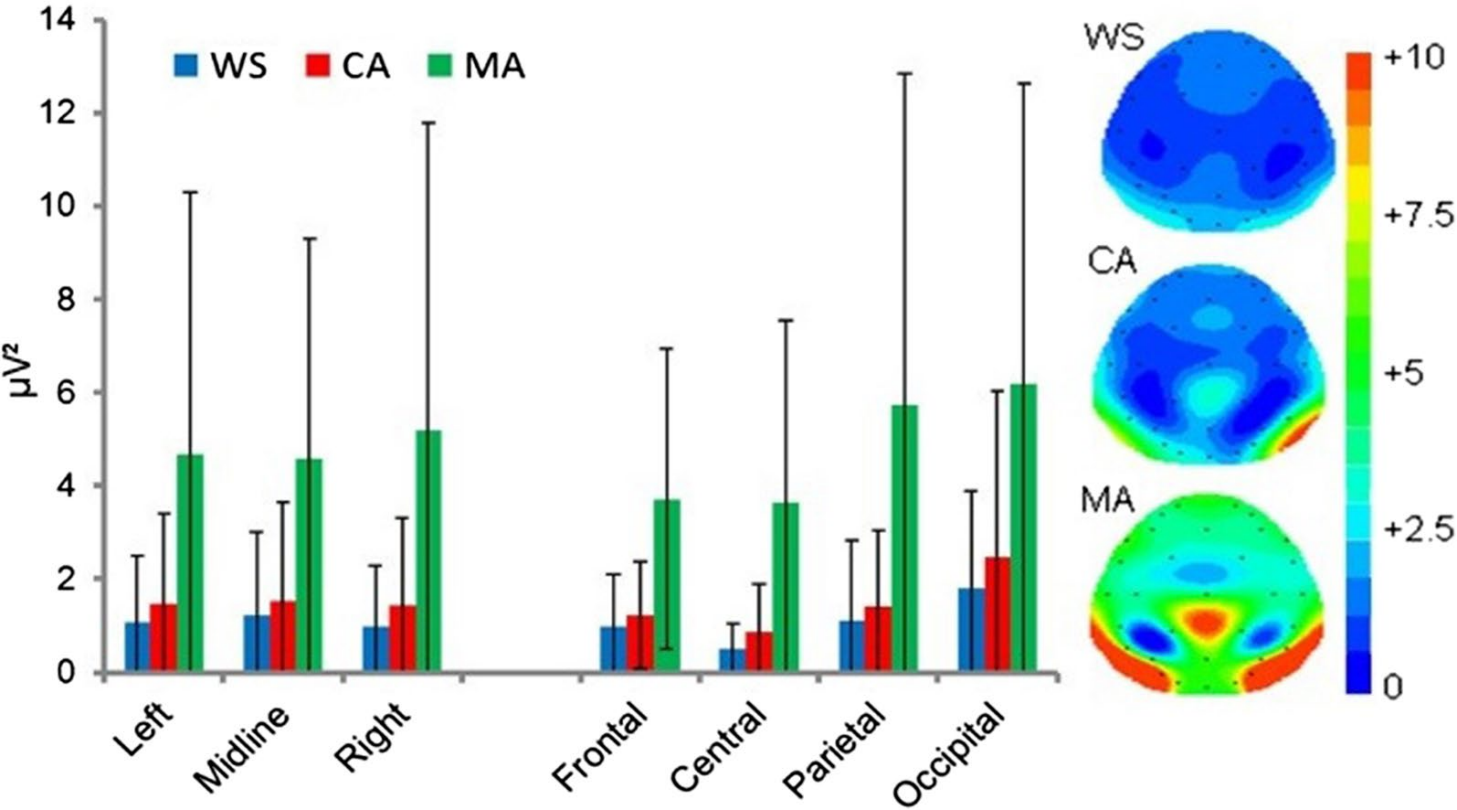
Mean absolute Eyes Open low-alpha power (µV^2^) and spectral mapping. Error bars represent SDs

The ANOVA identified a significant main effect of group [F(2,31) = 3.860, *p* = 0.032] on *α-low* power. Bonferroni corrected pairwise comparisons revealed numerically greater *α-low* power in the MA group that approached significance compared to the WS group (*p* = 0.051, *d* = 0.91), but not the CA group (*p* = 0.076, *d* = 0.79). There was no difference in *α-low* power between the WS and CA groups (*p* = 1.00, *d* = 0.22). The significant main effect of location [F(2.140,66.351) = 8.705, *p* < 0.001] was due to significantly greater occipital *α-low* power compared with both frontal and central locations (*p* ≤ 0.003) but not with the parietal location (*p* = 0.540). All other main and interaction effects were non-significant (*p* ≥ 0.104). See Fig. 4.

The effects sizes between the WS group and the MA and CA where *α-low* power maxima has been reported to be centred (PZ; [27]) were *d* = 0.91 (WS vs. MA) and *d* = 0.18 (WS vs. CA).

#### Upper-alpha band (α-high): 10–12.5 Hz

**Fig. 5:**
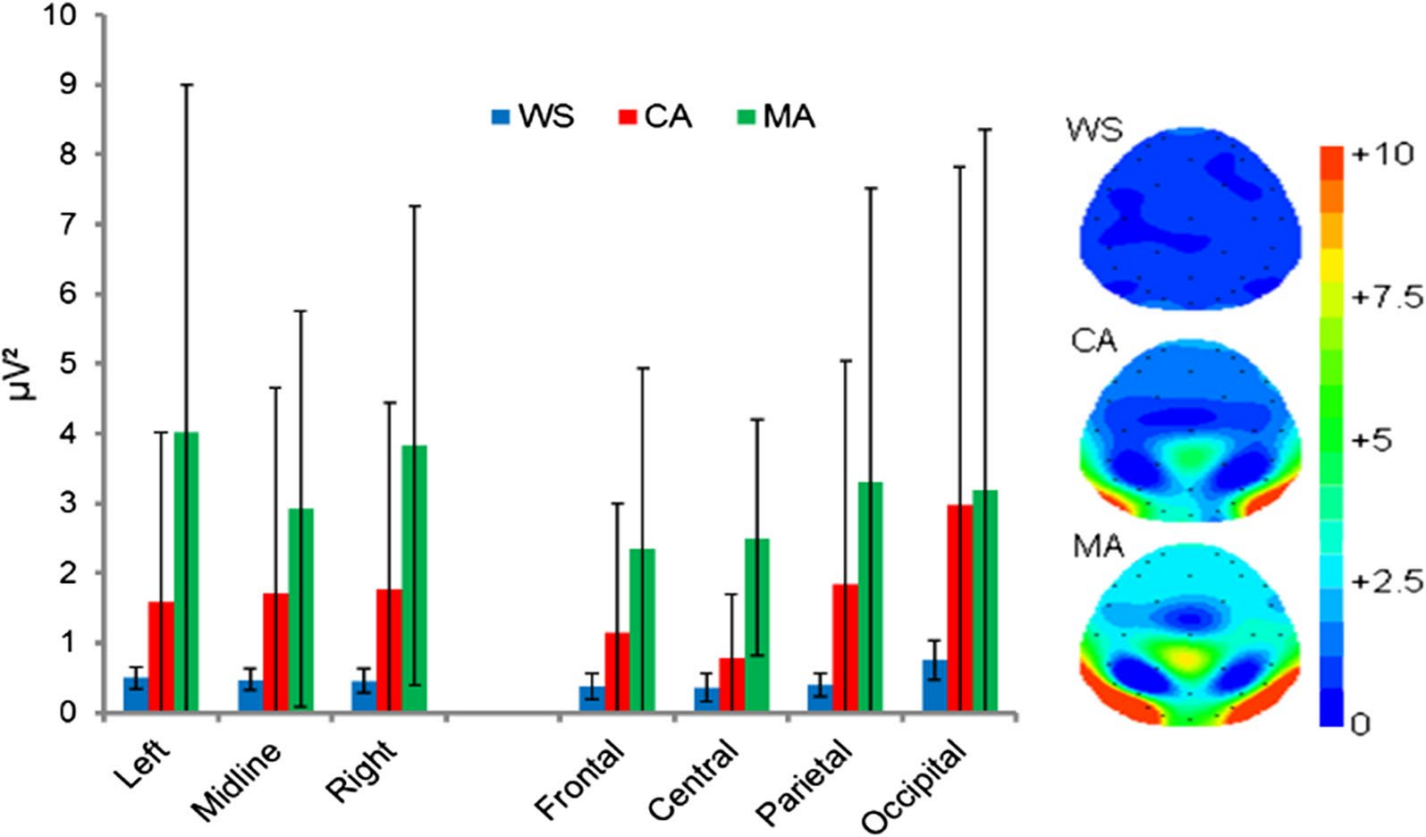
Mean absolute Eyes Open upper-alpha power (µV^2^) and spectral mapping. Error bars represent SDs

The ANOVA identified a significant main effect of group [F(2,31) = 3.788, *p* = 0.034]. Bonferroni corrected pairwise comparisons identified significantly greater *α-high* power in the MA group compared with the WS group (*p* = 0.031, *d* = 1.19). There was no difference in *α-high* power between the WS versus CA (*p* = 0.795, *d* = 0.64) and the CA versus MA (*p* = 0.277, *d* = 0.59) groups. Analyses also revealed a significant main effect of location [F(1.123,34.799) = 9.556, *p* = 0.003], due to significantly greater occipital *α-high* power compared with the frontal (*p* = 0.005), central (*p* = 0.036), and parietal (*p* = 0.003) locations. All other main effects and interactions were non-significant (*p* ≥ 0.090). See Fig. 5.

The effects sizes between the WS group and the MA and CA groups where *α-upper* power maxima has been reported to be centred (PZ; [27]) were *d* = 0.98 (WS vs. MA) and *d* = 0.64 (WS vs. CA).

#### Beta band (β): 13–29.5 Hz

**Fig. 6:**
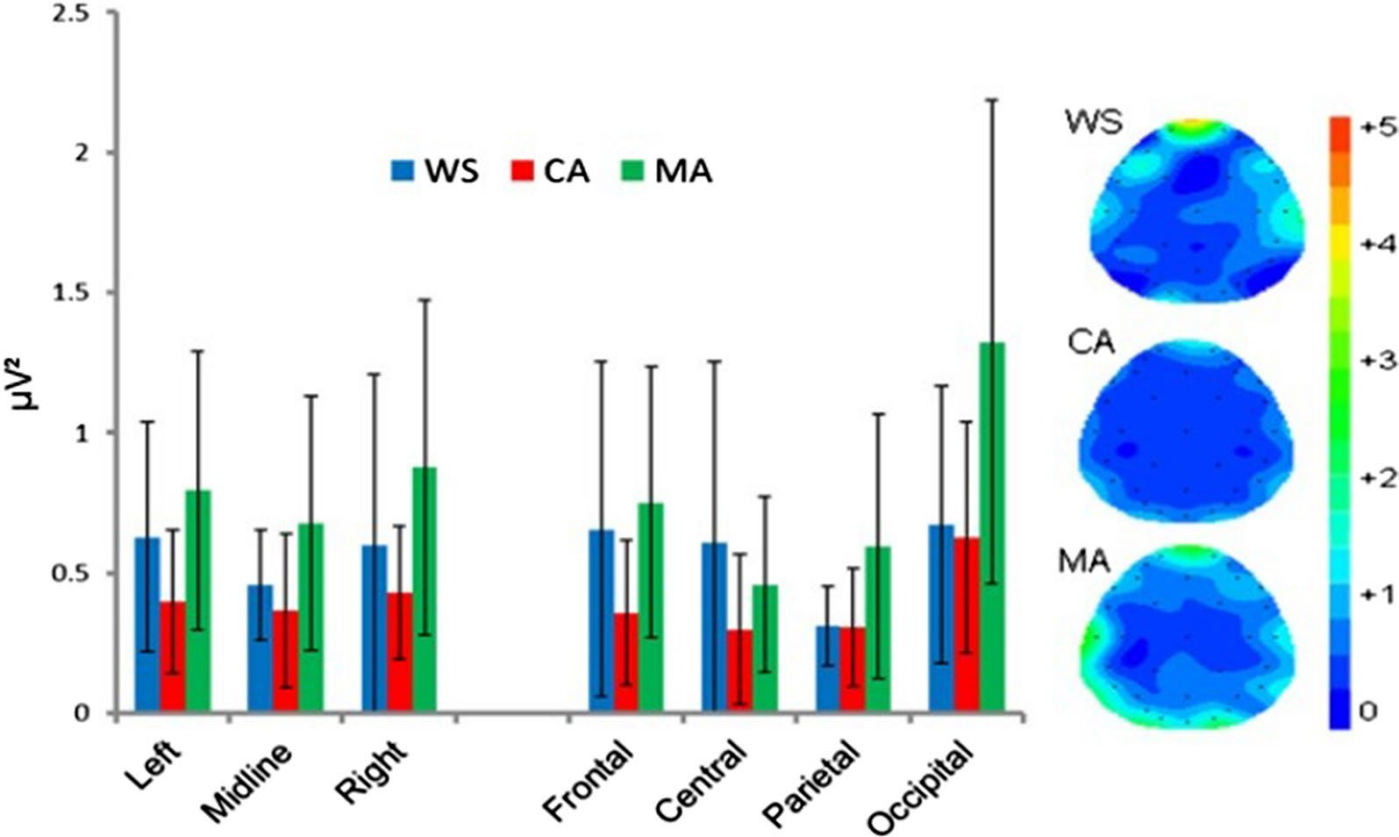
Mean absolute Eyes Open beta power (µV^2^) and spectral mapping. Error bars represent SDs

The main effect of group approached significance [F(2,31) = 3.249, *p* = 0.052] due to significantly greater β power in the MA group compared to the CA group (*p* = 0.048, *d* = 0.96), whereas differences between the WS vs. CA (*p* = 0.828, *d* = 0.59) and WS versus MA (*p* = 0.502, *d* = 0.53) groups were non-significant. The ANOVA revealed a significant main effect of location [F(2.119,65.694) = 13.523, *p* < 0.001]. Bonferroni corrected pairwise comparisons revealed significantly greater occipital β power than observed frontally (*p* = 0.034), centrally (*p* = 0.003), and parietally (*p* < 0.001). In contrast, parietal β power was significantly lower compared with the frontal location (*p* = 0.041). All other analyses were non-significant (*p* ≥ 0.520). See Fig. 6.

No main effect of hemisphere was observed, [F(1.395,43.231) = 3.063, *p* = 0.074] however analyses did reveal a significant group × location interaction [F(4.238,65.694) = 3.091, *p* = 0.020]. Examination of this interaction identified significant differences by group in the parietal [F(2,31) = 3.313, *p* = 0.050], and occipital [F(2,31) = 4.529, *p* = 0.019] locations. Bonferroni corrected pairwise comparisons revealed significantly greater occipital β power in the MA group compared with the CA group (*p* = 0.028, *d* = 1.03) group and approached significance compared with both the WS group (*p* = 0.054, *d* = 0.93). All other interaction analyses were non-significant (*p* ≥ 0.081).

The effects sizes between the WS group and the MA and CA groups where *Beta* power maxima has been reported to be centred (FZ; [77]) were *d* = 0.17 (WS vs. MA) and *d* = 0.64 (WS vs. CA).

### Summary

As observed in the EC results, the typically reported parieto-occipital maxima topographical distribution in the alpha band (full alpha and sub-bands) and the fronto-occipital maxima in the beta band mirrored the expected topographical distribution. Significant results by group were typically due to increased alpha and beta power in the MA group.

## Discussion

The current study examined the EEG spectral profiles of the alpha and beta bands in adults with WS, and how these support the attentional and inhibitory deficits reported in the literature (e.g. [9, 12, 13, 78]). The results of the current study are informative as, to date, there is no known published research which evaluates the spectral power profiles of adults with WS between EC and EO resting states. Analyses found that power in both the alpha (full and sub-bands) and beta bands observed in the WS group matched the topographical distributions observed in TD individuals during resting states. The analysis also confirms the WS group’s profile is not reflective of their verbal mental age; therefore our evaluation of the data focuses primarily on the WS and CA groups’ results.

Overall, during the EC condition, all groups reported a profile characterised by a posterior topographical distribution as previously reported [27, 52, 53]. Upper-alpha was characterised by an occipital maximum as expected [27, 54] whist full- alpha and low-alpha were characterised by an occipito-parietal distribution [79]. Opening the eyes (EO) resulted in an overall attenuation of cortical activity in both alpha and beta bands in all groups as expected based on the evidence from TD individuals [27, 52, 53], and from the limited developmental disorder literature (e.g. ASD, [61]; ADHD, [57]). Inspection of the full- and sub-bands of alpha identified similar topographical distributions by location as observed during EC. There were no differences in beta power by hemisphere on opening the eyes, however all groups reported an occipital maximum [52, 53, 55].

When considering results by group, overall, the differences were dominated by significantly greater alpha power in the MA group compared with the WS group during both the EC and EO conditions, and significantly greater beta in the MA group compared with the CA group during the EO condition. These are likely reflective of age-associated differences in oscillatory firing rates due to neuronal maturation [80, 81]. In contrast, the differences between the WS and CA groups were non-significant across all frequencies, though the WS group consistently reported numerically lower power in the full-alpha band and alpha sub-bands in both conditions. The lack of statistical significance prevents interpretation of the observed attenuated alpha power in the WS group, however attenuated full- and low-alpha compared to the CA and MA groups was predicted based on the existing literature with other developmental disorders with attentional deficiencies including FXS [59], DS [56], and ADHD [57, 82, 83]. In WS, significantly attenuated alpha power has been observed in research using combined EC/EO data [67], and in sleep states [64, 65]. In ADHD, attenuated alpha power during resting states is thought to reflect an ongoing state of cortical hyper-arousal even in the absence of cognitive processing [48]. This is of notable interest due to the atypical behavioural attentional and inhibitory profile associated with WS [9, 84–86] and warrants further investigation with greater sample sizes in order to establish whether this can replicated and can be supported statistically. Furthermore, future research needs to take a cross-syndrome approach to identify whether this a deficit specific to neurodevelopmental disorders or indicative of atypical attentional control in general.

In the beta band, there were no group differences between the WS and the CA controls during both conditions, which was not expected. Whilst previous research demonstrates greater beta power in WS during sleep [64], cortical and subcortical activity differs between resting- and sleep states [87–89]. Thus, the hypothesis of attenuated beta was guided by the ADHD literature, in which attenuated beta is widely documented as part of its EEG profile (e.g. [58]). Other developmental disorders also report contradictory findings; beta is attenuated in DS during EC [56], comparable to controls in FXS [59], but inconsistent in ASD [61]. Whilst these differences make interpretation of the functional significance of beta in developmental disorders more complicated, the comparable beta power between the WS and CA groups is informative here. Indeed, these comparisons across groups are highly informative for the development of syndrome-specific theories. The beta band is typically associated with visuo-attentional processes [35–37] and linked with motor control [90]. It has been demonstrated that behavioural performance (hit rates) in WS is comparable to controls during conditions of low attentional demands (e.g. [12, 14]), whereas performance is impaired compared to controls when attentional demands are great (e.g. [78]). [Of note, the emphasis here is on level of task difficulty, as greater RT in all of the aforementioned studies was indicative of general attentional deficits in this group of adults with WS.] Thus, the comparable levels in beta power between the WS group and CA controls reported here suggest that the small sample of individuals with WS recruited for this study have a profile of resting-state cortical activation commensurate with successful attentional processing and motor control. Future research paradigms should therefore focus on beta power during resting states, and also during low- and high-attentional processing in a much larger sample of individuals with WS, in order to elucidate (a) whether the pattern here can be replicated, (b) at what stage in cognitive processing atypicalities (if any) in beta power manifest, and (c) how these sub-serve their attentional deficits reported behaviourally. A further important area of research would be to investigate the alpha/beta ratio in WS, and its impact during attentional processing (cf. [91]). Currently, the dearth of research employing EEG methodology in WS makes interpretation of the current data more challenging.

The aforementioned questions may be in part answered by investigating the role of variability in the WS EEG profile. The issue of variability is widely documented in the WS, with high levels of variability typically associated with WS behavioural and cognitive phenotypes [4, 92]. Visual inspection of the current data noted an inverse pattern of variance, notably in the alpha band, with high variability in the control groups and low variability in the WS group. Thus, the lack of statistical significance between groups in the current study may be in part due to the high levels of variability identified in the alpha bands of the CA group; though we emphasise this is a highly tentative suggestion and should be addressed with caution as no statistical analyses were conducted. Low variability in the WS group was more apparent in the upper-alpha band of both conditions, and low-alpha during EC; whereas variability in low-alpha during the EO condition was similar to that observed in the beta band. Though the functional significance is not clearly defined, low-alpha and beta are both associated with attentional processes [27, 36, 37]. Therefore, this pattern of variability may be indicative of a dissociation in WS between the EO resting-state alpha oscillations which sub-serve general attentional processes and those with a greater functional association with more specialised cognitive processes. This clearly requires further empirical investigation and with much larger sample sizes before any interpretation can be made as this contradicts the heterogeneity typically associated with WS. However, this phenomenon of reduced variability has also been previously reported in EEG research with ADHD adults [57], who also observed significantly less variability in the alpha band in adults with ADHD compared with healthy controls.

A final notable finding of the study relates to the mental age-matching procedure. The issue of appropriate control group matching has been discussed throughout the developmental disorder literature (see [72]). As noted, the MA group consistently reported numerically greater alpha and beta power in both the EC and EO conditions compared with the WS and CA groups. In most analyses this was significantly greater than the WS group, but notably during the EO condition the MA group reported significantly greater beta power than the CA group. A caveat when comparing EEG profiles between adults and children are differences in oscillatory firing rates, as these are typically faster in children than in adults (for a discussion, see [81]). Thus, frequency distributions between adults and children may not be comparable as the developmental profile of EEG oscillations is not complete until early adulthood [80]. The children in the MA sample here were aged from 8 to 16 years of age with the majority of participants aged ~ 12 years old, thus including verbal mental age-matched controls is not overly informative (see [93] for an extensive study on developmental and child pathological comparison). Rather than mental-age matching, comparison with an atypically developing cohort such as ADHD would be more beneficial in order to elucidate whether group differences are syndrome-specific, or due to atypical development in general.

## Conclusion

In conclusion, to date the current study is the first known research endeavour to evaluate the oscillatory profile of the alpha and beta bands in WS during EC and EO resting states separately. The study found no significant difference in full-alpha power or alpha sub-bands between our WS sample and the CA/MA control groups; however the numerically lower power observed in the WS group is similar with other developmental disorders characterised by attentional/inhibitory deficits and requires further empirical investigation. In contrast, the comparable beta power between WS and CA groups during both EC/EO conditions suggests that their baseline EEG signature is commensurate with successful attentional processing, though this needs to be interpreted with caution due to the small sample size. Notably, the unusual trend for low variability in the EEG signature of the WS group is a novel observation as this contradicts the heterogeneity typically observed behaviourally. This clearly also warrants further investigation with both larger sample sizes including a group of WS individuals with a more variable attentional profile, and with more detailed inspection of functional connectivity, in order to clarify the role of EEG variability in the cognitive and behavioural profile associated with the syndrome.

## Data Availability

The datasets generated and analysed during the current study are available in the Northumbria University research repository. (https://doi.org/10.25398/rd.northumbria.12254054).

## Abbreviations

ADHD: Attention deficit hyperactivity disorder
ANOVA: Analysis of variance
ASD: Autism spectrum disorder
BPVS: British Picture Vocabulary Scale
CA: Chronologically age-matched
CPT: Continuous performance task
DS: Down syndrome
EC: Eyes closed
EEG: Electroencephalography
EHI: Edinburgh handedness inventory
EO: Eyes open
ERP: Event related potential
FISH: Fluorescence in situ hybridisation
fMRI: Functional magnetic resonance imaging
FXS: Fragile X syndrome
MA: Mental age-matched
RT: Reaction time
TD: Typically developing
WS: Williams syndrome
